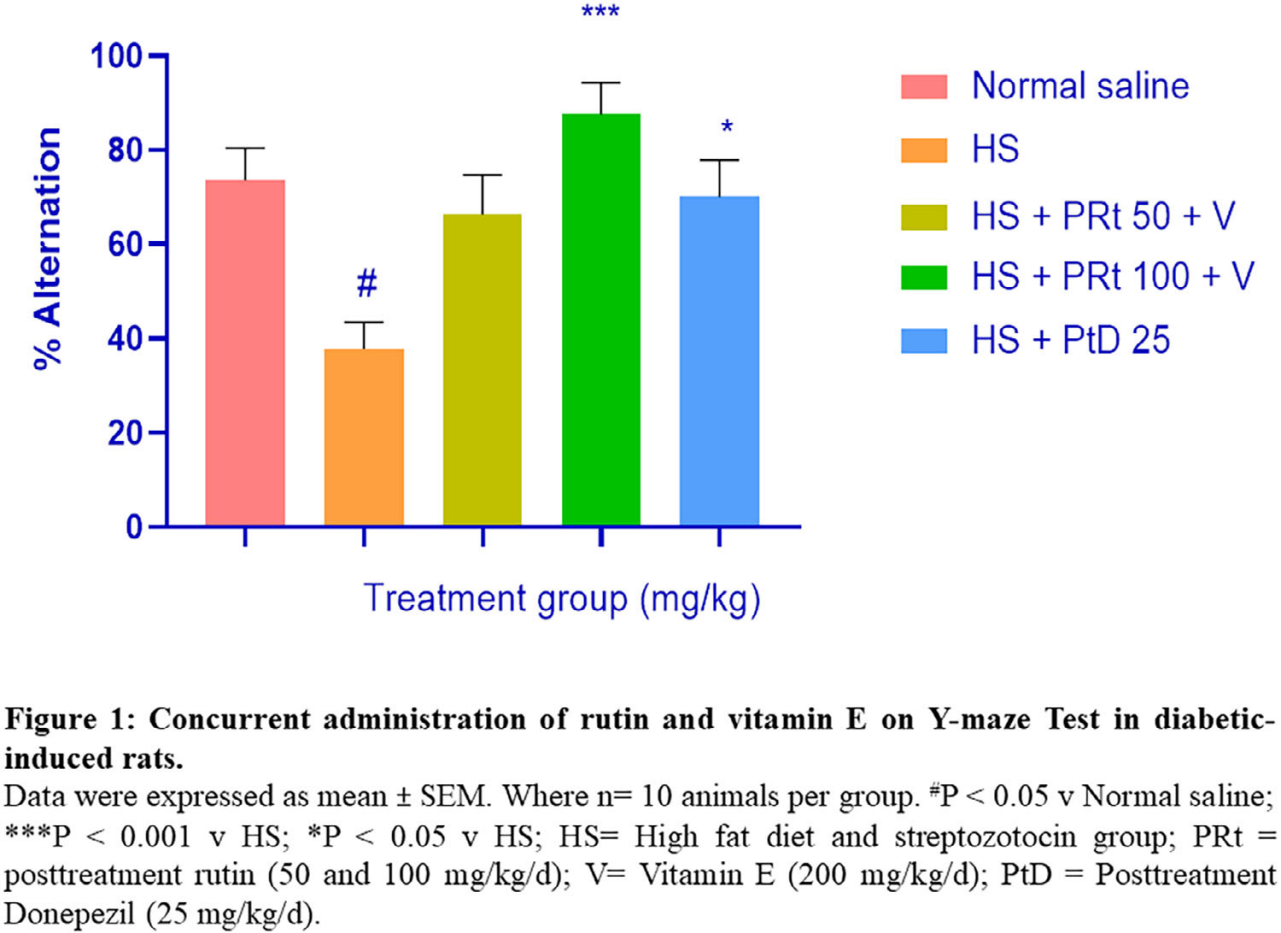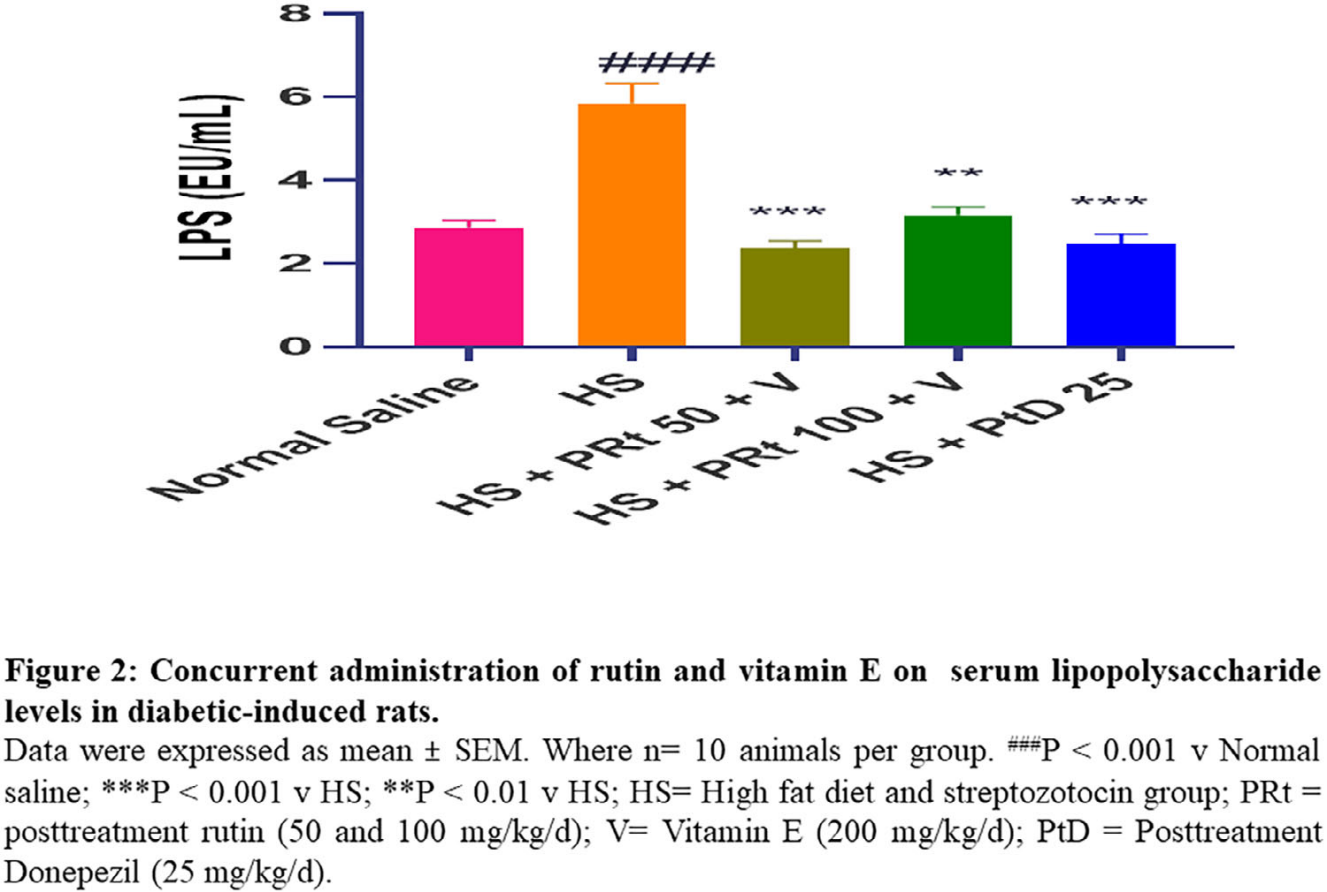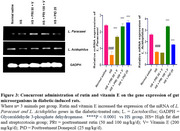# Co‐administering rutin and vitamin E alleviates diabetic memory impairment associated with regulating gut microbiota

**DOI:** 10.1002/alz70859_097101

**Published:** 2025-12-25

**Authors:** Juliet Nnenda Olayinka, Racheal Iyewande Akinnagbe, Hafsat Ayomide Salihu, Muhammed Zubairu Adamu, Joyce Eve Ngue, Erica Sewuese Ukura, Micheal Uche Uko, Lily Oghenevovwero Otomewo, Adedamola Adediran Fafure, Abiola Oluwatosin Obisesan, Olusegun Adebayo Adeoluwa, Stephen Olushola Saka, Bright Christian Chukwu, Benjamin Olushola Omiyale, Adams Olalekan Omoaghe, Raymond Iduojemu Ozolua

**Affiliations:** ^1^ Afe Babalola University, Ado‐Ekiti, Ekiti State Nigeria; ^2^ university of Benin, Benin City Nigeria; ^3^ Afe Babalola University, Ado‐Ekiti, Ekiti State Nigeria; ^4^ Afe Babalola University, Ado‐Ekiti, Ekiti State, Ekiti State Nigeria; ^5^ Afe‐Babalola University, Ado‐Ekiti, Ekiti State Nigeria; ^6^ Afe Babalola University, Ado‐Ekiti, Ekiti State Nigeria; ^7^ University of Benin, Benin city, Edo State Nigeria

## Abstract

**Background:**

Type 2 diabetes mellitus (T2DM) induces memory loss by triggering inflammation and gut dysbiosis. Evidence from research has shown that these pathologic effects of T2DM may be attenuated through the use of antioxidants. Rutin and vitamin E co‐administration may serve as effective therapeutic strategies since both antioxidants have shown antihyperglycemic and memory‐enhancing effects in previous studies. Here we investigated how gut microbiota regulation by rutin and vitamin E co‐administration prevented T2DM‐induced pathologic processes and prevented T2DM‐induced memory impairment.

**Method:**

Five groups of 50 male rats (*n* = 10) were administered HFD for 28 days, excluding the normal saline group. Streptozotocin (35 mg/kg intraperitoneally) was administered twice. Weekly blood glucose and weight were recorded. For three weeks, rats were concurrently administered rutin (50‐100 mg/kg/d, orally) with vitamin E (200 mg/kg/d) or donepezil (25 mg/kg/d). Fecal samples collected after the final treatment were utilized to analyze short‐chain fatty acids (SCFA) utilizing high‐performance liquid chromatography and mRNA expression of the genes of *Lactobacillus (L.) paracasei* and *L. acidophilus* using the polymerase chain reaction technique. Memory function (Y‐maze) was performed. Blood was collected from the heart to estimate lipopolyssacharide (LPS), insulin resistance, tumor necrosis factor‐alpha (TNF‐α), and interleukin‐6 (IL‐6) levels using an enzyme‐linked immunosorbent assay. The colon and the cornus ammonis 1 of the hippocampus were harvested for histological and immunofluorescent assays (to determine the expression of tight junction protein (occludin) in the colon and brain).

**Results:**

Administering rutin and vitamin E to diabetic rats improved memory function, reduced weight, glucose, insulin resistance, TNF‐α, LPS, and IL‐6 levels, and increased SCFA‐acetic, propionic, isobutyric, adipic, butyric, valeric, and isovaleric acid levels compared to the diabetic rats. Compared to diabetic rats, rutin and vitamin E co‐administration reduced colon lesions and neuronal loss, restored colon and blood‐brain barrier tight junction protein‐occludin, and increased the mRNA expression of *L. paracasei* and *L. acidophilus* genes.

**Conclusion:**

These findings suggest that the therapeutic effect of rutin and vitamin E may be associated with the regulation of the gut microbiota, and these drugs might serve as promising therapeutic strategies in managing memory loss in T2DM.